# Web-Based Health Information Seeking Among Students at Kuwait University: Cross-Sectional Survey Study

**DOI:** 10.2196/14327

**Published:** 2019-10-31

**Authors:** Hasan Ashkanani, Rabab Asery, Fajer Bokubar, Noor AlAli, Shahad Mubarak, Ali Buabbas, Abdullah Almajran

**Affiliations:** 1 Faculty of Medicine Kuwait University Jabriyah Kuwait; 2 Department of Community Medicine Faculty of Medicine Kuwait University Jabriyah Kuwait

**Keywords:** Kuwait, online, health care

## Abstract

**Background:**

Owing to the revolution in technology, the internet has become an important aspect of people’s lives. Modern technology is enabling people from diverse educational backgrounds to use the internet for several purposes, one of which is health information seeking. Recently, Web-based health information has become more popular among patients all over the world and among the general public.

**Objective:**

This study aimed to investigate the use of Web-based health resources among undergraduate students from different faculties at Kuwait University.

**Methods:**

The study employed a cross-sectional design with students selected from 8 faculties of Kuwait University, 4 faculties of Literature and 4 faculties of Science. Data were collected using structured questionnaires, and analysis was done using a chi-square test and binary logistic regression to determine the factors associated with seeking health information on the Web.

**Results:**

The sample size obtained was 1132 with a response rate of 90.27% (1132/1254). Overall, the prevalence of students seeking Web-based health information was 92.66%. (1049/1132) The most significant factors associated with seeking health information on the Web were age, gender, faculty, year of study, primary source of internet, and level of experience with internet use. In total, 90.0% (325/361) of students who were aged older than 21 years used Web-based health information compared with 82.8% (275/332) of those who were aged 18 years. In addition, female students showed a higher prevalence (829/934, 88.8%) of Web-based health information seeking than males (210/270, 77.8%). Students who majored in faculties of Science were more likely to seek health information than those who majored in faculties of Literature. All the differences found in the study were statistically significant (*P*<.05).

**Conclusions:**

The study concluded that many people use the internet for seeking health information. Sociodemographic factors have a significant association with Web-based health information seeking. Therefore, doctors must educate the public about the health information websites that can be trusted.

## Introduction

“The internet is a global network that enables computers and other communication gadgets to communicate directly and transparently” [1]. Owing to the revolution in technology, the internet has become an important aspect of people’s lives. Modern technology is enabling people from diverse educational backgrounds to use the internet for several purposes, one of which is health information seeking. People use the internet to search for several health-related topics, including facts about different diseases, diagnostic criteria, treatment methods, and side-effects of various medications.

Studies show that Web-based health information has become more popular among patients all over the world and among the general public. According to a study conducted in 2008, there was a 38% increase in the number of health-related websites in the Eastern Mediterranean Region [2]. Health information seekers can be defined as “internet users who search online for information about health topics whether they are acting as consumers, care givers, or e-patients” [3].

Previous studies concluded that most health-related internet searches are conducted to look for information related to a specific medical condition, both before and after seeking a consultation from a health care professional. This development was very prominent within the last 10 to 15 years. Concerns about information found on the Web include the quality of information found, whether the information would improve the patient’s health, and whether the patient-doctor relationship will improve as patients become more informed or be damaged if doctors have difficulty when they recommend changes to patients with preconceived notions [4]. Doctors and the organizations that support them have to fully understand their role in the health revolution that was the result of Web-based health information seeking, both of which must improve their awareness of the patients and their needs. [5].

It is thought that the use of the internet by patients will raise their awareness about their conditions, whereas its use by doctors will assist their ability to make evidence-based decisions. A study conducted in the United States concluded that the challenge faced by the doctors is translating scientific advances into meaningful advice for clinical care [6].

As a source of information, the internet has several advantages over traditional sources. These advantages include easy access to information, frequently updated material, confidentiality, and interactivity, which leads to better understanding. Web-based resources of health information used to not be readily available for public consumption, as the internet was not known as a destination for medical information. Therefore, patients did not have any health information, and their roles were limited to passive roles that consisted of complying to the orders of their health care providers. However, with the rise of innumerable websites and apps that provide health information in a simplified manner, there was a shift in the paradigm, and in recent years, patients have been opting for a more active role in seeking health care. Using Web-based resources for health purposes empowers the patients to seek a more active role [4].

In spite of the various advantages of the internet, it can also be harmful when consumers use it without evaluation of the quality of the information sought. This can lead to misinterpretation of the information, affecting health behavior and outcomes [4,7].

A previous study conducted in 2003 had concluded that 61% of citizens of the United States who use the internet have searched for health information on the Web compared with 58.4% of citizens in Saudi Arabia [8,9]. A study conducted in Kuwait found that 93.2% of patients use the internet for one or more purposes and that 62.9% of these patients use it for seeking health-related information. In addition, it was concluded that gender, nationality, level of education, and using a computer at work were significant factors for obtaining health information on the Web [10]. This is consistent with another study conducted in Saudi Arabia, which showed that gender, education, employment, and income affect Web-based health information seeking [8].

Although there has been an increase in the use of the internet in health care, little research has been conducted to study the impact that the availability and integrity of health care information on the internet has on users. This study aimed to investigate the use of Web-based health resources among undergraduate students at Kuwait University.

The objective of the study was 3-fold: (1) to determine the prevalence of Web-based health information seeking among students at Kuwait University; (2) to determine the influence of the information found on the Web on the attitudes of students at Kuwait University; and (3) to identify the association between the sociodemographic factors of the students and their health information–seeking patterns.

## Methods

### Research Setting

Kuwait University is the first public university in the State of Kuwait and was established in 1966. Its vision is to provide world-class education, and it is committed to advancing, preserving, and disseminating knowledge, in addition to preparing educated, enlightened, and qualified human resources to realize the society’s developmental needs. It consists of 17 faculties, divided into faculties of Literature and faculties of Science.

### Study Design and Study Sample

This cross-sectional study was conducted among undergraduate students at Kuwait University across various faculties of the university. The study sample included 8 randomly selected faculties, stratified as 4 faculties of Literature and 4 faculties of Science. The selected faculties of Science were Medicine, Dentistry, Pharmacy, and Science, and the selected faculties of Literature were Law, Sharia and Islamic Studies, Education, and Business Administration (the total population of the 8 faculties was 19,359 students). A purposive convenient sampling procedure was used for students involved in the study, and the instrument used was a validated questionnaire. Students were approached in between classes and during their breaks, and participation was voluntary. The study included students aged between 18 and 25 years. Any student aged younger than 18 years or older than 25 years was excluded from the sample population. A total of 1253 questionnaires were collected during this period, and the response rate was 99.12% (1242/1253).

### Ethical Approval

A Human Subject Form describing the ethical aspects of the study was approved by the Faculty of Medicine Ethical Committee, and an informed consent sheet was prepared, both ensuring protection of the participant’s autonomy, beneficence, and justice. There were no risks to participation in this study, and benefits of the study were explained to each participant while obtaining their informed consent. Rejection rate as per faculty is provided in Multimedia Appendix 1.

The study protocol and data collecting instruments were reviewed and approved by the Health Sciences Ethical Committee for Student Research. In addition, the permission of the vice deans of each faculty and university in which the study was conducted was sought. The objectives of the study were explained to the students before acquiring their written consent. The anonymous questionnaires that were used assured privacy and confidentiality of the data collected. All students participated voluntarily and understood that they had the right to refuse to partake in the study at any time without any academic penalty (Multimedia Appendix 2).

### Study Questionnaire and Pretesting

A validated questionnaire from a study conducted in Ghana was used to collect data from the students [1]. However, the questionnaire was modified to better suit the students at Kuwait University. The questionnaire was translated and distributed in both English and Arabic for convenience. It consisted of 31 questions divided into 7 sections (Multimedia Appendix 3). The first section consisted of questions regarding students’ basic sociodemographic characteristics (age, gender, and nationality). The other sections were validated tools to assess students’ (1) access and use of Web-based resources; (2) use of devices, apps, and platforms; (3) use of Web-based resources for health purposes; (4) usage of the information found on the Web; (5) rating of the legitimacy of health information sought using Web-based sources; and (6) attitudes and beliefs about the use of Web-based resources for finding health information.

The questionnaire was reviewed by 2 faculty members, and their feedback was incorporated during revision of the questionnaire. The questionnaire was pretested on 10 students similar to the target population, and both the English and Arabic versions were revised as required after pretesting. The questionnaires took on average 5 to 10 min to complete.

### Data Collection

All the data were collected in a 2-week period (from March 29 to April 14, 2018). A total of 1253 questionnaires were collected during this period.

### Statistical Analysis

Data were entered, cleaned, and analyzed using the Statistical Package for the Social Sciences version 25 (IBM). Univariate analysis was performed to calculate percentages, frequencies, means, and standard deviations. Significant associations between dependent and independent categorical variables were tested using the Pearson chi-square test. The binary logistic regression model was used to adjust the odds ratio for potential cofounding variables. Age, gender, and level of experience with internet use were the variables included as covariates in the model for adjustment as they were significantly associated with the score in the crude analysis. A *P* value of ≤.05 and 95% CI were considered to be the levels of significance.

## Results

Table 1 presents the characteristics of the study participants. The variables are gender, age, faculty, nationality, year of study, frequency of Web use, main device used to access the internet, type of internet used on campus, primary source of internet access, and the level of experience with Web use. The study participants were 1132 students from 8 different faculties. The age of the participants varied from 18 to 25 years with an average of 20 years.

Most of the respondents were Kuwaitis (993/1132, 87.72%) and females (880/1132, 77.73%). Most of the students were from the Faculty of Business Administration (192/1132, 16.96%) with only few from the Faculty of Pharmacy (76/1132, 6.71%). Students in their first year of study were the most (274/1132, 24.20%), and students in their seventh year were the least (14/1132, 1.23%). The students most often accessed the Web daily (1063/1132, 93.90%) using smartphones as the main device (1089/1132, 96.20%). Mobile data was used the most on campus (726/1132, 64.13%), with only few students using the internet cable (3/1132, 0.26%). In addition, the primary source of internet use was a personal device and the least was the university computer laboratory. Most students were very experienced with Web use (623/1132, 55.03%), whereas few students rated themselves as not experienced (11/1132, 0.97%).

The sociodemographic status of the students is presented in Table 1. The mean age of the students was 20.7 years, and most of the students who participated in the study were Kuwaitis (993/1132, 87.72%) and females (880/1132, 77.73%). Approximately 16.96% (192/1132) of the students were from the Faculty of Business Administration, whereas 6.71% (76/1132) of the students were from the Faculty of Pharmacy. Almost 24.20% (274/1132) of the students in the study were freshmen. Most of the students used the Web daily (1063/1132, 93.90%). In addition, 96.20% (1089/1132) of the students used their smartphones as the main device while accessing the Web. Mobile data was the most used type of internet on campus (726/1132, 64.13%). Most of the students used their personal device as the primary source of internet (972/1132, 85.86%). Almost half of the students (1132/623, 55.03%) were very experienced with using the internet.

**Table 1:** Sociodemographic characteristics of the study sample (N=1132).

Variables		Values
Age (years), mean (SD)		20 (1.919)
**Nationality, n (%)**		
	Kuwaiti	993 (87.72)
	Non-Kuwaiti Arab	123 (10.86)
	Non-Kuwaiti non-Arab	14 (1.23)
	Others	2 (0.17)
**Gender, n (%)**		
	Male	252 (22.26)
	Female	880 (77.73)
**Faculty, n (%)**		
	Medicine	190 (16.78)
	Pharmacy	76 (6.71)
	Dentistry	60 (5.30)
	Science	170 (15.01)
	Education	163 (14.39)
	Business administration	192 (16.96)
	Sharia and Islamic studies	173 (15.28)
	Law	108 (9.54)
**Year of study, n (%)**		
	First	274 (24.20)
	Second	182 (16.07)
	Third	268 (23.67)
	Fourth	223 (19.69)
	Fifth	117 (10.33)
	Sixth	47 (4.15)
	Seventh	14 (1.23)
**How often do you use the Web (days), n (%)**		
	Daily	1063 (93.90)
	1-3	23 (2.03)
	4-7	44 (3.88)
	Never	2 (0.17)
**Main device used, n (%)**		
	Smartphone	1089 (96.20)
	Laptop	23 (2.03)
	Tablet	14 (1.23)
	Desktop computer	6 (0.53)
**Types of internet used on campus, n (%)**		
	Mobile data	726 (64.13)
	Campus Wi-Fi	71 (6.27)
	Internet cable	3 (0.26)
	Personal Wi-Fi	332 (29.32)
**Primary source of internet use, n (%)**		
	University computer laboratories	29 (2.56)
	University library	34 (3.00)
	Home	97 (8.56)
	Personal device	972 (85.86)
**Level of experience with internet use, n (%)**		
	Very experienced	623 (55.03)
	Fairly experienced	498 (43.99)
	Not experienced	11 (0.97)

Table 2 shows significant associations between students who seek Web-based health information (n=1126) and age, gender, faculty, year of study, primary source of internet, and level of experience with *P* values <.05 with the exception of nationality, frequency of using the internet, main device used, type of internet used on campus, and internet use in years. Significant associations were identified as *P*<.05.

The association between the sociodemographic factors of the students and Web-based health information seeking is presented in Table 2. In total, 90.0% (325/361) of the older age group (more than 21 years of age) used the Web for health information seeking compared with 82.8% (275/332) of the relatively young age group (18-19 years of age). The association between age and Web-based health information seeking is significant (*P*=.006). However, there was an insignificant association between the students’ nationality and seeking Web-based health information. Female students showed higher prevalence of health information seeking (829/934, 88.8%) than their male counterparts (210/270, 77.8%; *P*<.001).

The association between faculties and Web-based health information seeking was found to be statistically significant (*P*=.01). The highest prevalence of seeking health information on the Web was among students of the faculties of Pharmacy (79/83, 95.2%), Dentistry (63/68, 92.6%), Medicine (178/198, 89.9%), and Science (167/191, 87.4%). On the contrary, the faculties that had a lower prevalence were faculties of Sharia and Islamic studies (152/181, 84.0%), Business Administration (167/199, 83.9%), Law (98/118, 83.1%), and Education (137/169, 81.1%). Most of the students who used the Web for health information were from the sixth year (54/58, 93.1%), whereas first year students were the least to use the Web for retrieving health information (233/284, 82.0%; *P* value=.003).

In addition, the study showed no significant association between the frequency of Web use, main device used, and the type of internet used on campus with Web-based health information seeking. In addition, there was no significant association between internet use in years with Web-based health information seeking.

Most of the students (874/997, 87.7%) used personal devices as their primary source of internet, whereas the university library was the least used source (32/44, 72.7%). About 88.6% (576/650) of the students were very experienced with internet use, whereas 79% (15/19) were not experienced. Both associations between the primary source of internet and level of experience and Web-based health information seeking were found to be significant (*P* values=.005 and .008, respectively). After adjusting for odds ratio, nationality, faculty, year of study, frequency of internet use, main device used, type of internet used on campus, and primary source of internet were found insignificant, whereas age, gender, and level of experience were found to be significant.

Table 3 shows significant associations between students who use Web-based health information seeking and apps, search engines, YouTube, emails to doctors, and websites (*P*<.05). The students’ use of devices, apps, and platforms to seek health information is demonstrated in Table 3. The study found that students use search engines, for example, Google, Bing, and Yahoo, the most to seek health information (954/1091, 87.44%). YouTube (882/1007, 87.58%) and apps for smartphones and tablets (93/104, 89.4%) were found to be the second and third most used to seek health information, respectively. The least used method to seek health information was email to doctors (282/312, 90.4%). Twitter was the most used (314/358, 87.7%) social media platform in terms of seeking health information, whereas Facebook was the least used (10/837, 1.1%) social media platform.

**Table 2:** Relationship between sociodemographics and Web-based health information seeking (N=1126).

Variables		Total, N	Web-based health information seeking, n (%^a^)	*P* value^b^
**Age (years)**				
	18-19	332	275 (82.8)	.006^c^
	20-21	433	371 (85.7)	.006^c^
	More than 21	361	325 (90.0)	.006^c^
**Nationality**				
	Kuwaiti	1045	898 (85.93)	.77^d^
	Non-Kuwaiti Arab	132	117 (88.6)	.77^d^
	Non-Kuwaiti non-Arab	17	15 (88.2)	.77^d^
	Others	2	2 (100.0)	.77^d^
**Main device used**				
	Smartphone	1123	971 (85.77)	.69^d^
	Laptop	38	32 (84.2)	.69^d^
	Tablet	29	25 (86.2)	.69^d^
	Desktop computer	12	9 (75.0)	.69^d^
**Type of internet used on campus**				
	Mobile data	743	644 (86.7)	.75^d^
	Campus Wi-Fi	91	76 (83.5)	.75^d^
	Internet cable	3	3 (100.0)	.75^d^
	Personal Wi-Fi	370	318 (85.9)	.75^d^
**Primary source of internet**				
	University computer laboratory	39	30 (76.9)	.005^d^
	University library	44	32 (72.7)	.005^d^
	Home	126	104 (82.5)	.005^d^
	Personal device	997	874 (87.7)	.005^d^
**Gender**				
	Male	270	210 (77.8)	<.001^d^
	Female	934	829 (88.8)	<.001^d^
**Faculty**				
	Medicine	198	178 (89.9)	.01^d^
	Pharmacy	83	79 (95.2)	.01^d^
	Dentistry	68	63 (92.6)	.01^d^
	Science	191	167 (87.4)	.01^d^
	Education	169	137 (81.1)	.01^d^
	Business administration	199	167 (83.9)	.01^d^
	Sharia and Islamic studies	181	152 (84.0)	.01^d^
	Law	118	98 (83.1)	.01^d^
**How often do you use the internet**				
	Daily	1079	938 (86.93)	.1^d^
	1-3 days	53	40 (75.5)	.1^d^
	4-7 days	73	61 (83.6)	.1^d^
	Never	1	1 (100.0)	.1^d^
**Year of study**				
	First	284	233 (82.0)	.003^c^
	Second	199	168 (84.4)	.003^c^
	Third	277	241 (87.0)	.003^c^
	Fourth	234	207 (88.5)	.003^c^
	Fifth	127	112 (88.2)	.003^c^
	Sixth	58	54 (93.1)	.003^c^
	Seventh	25	23 (92.0)	.003^c^
**Level of experience with internet use**				
	Very experienced	650	576 (88.6)	.008^c^
	Fairly experienced	537	449 (83.6)	.008^c^
	Not experienced	19	15 (79)	.008^c^
**Internet use (years)**				
	Less than 1	167	161 (96.4)	.11^d^
	1-2	221	217 (98.2)	.11^d^
	3-4	252	251 (99.6)	.11^d^
	More than 4	406	398 (98.0)	.11^d^

^a^Row percentages.

^b^Significance: *P*<.05.

^c^Chi-square test for trend.

^d^Pearson chi-square test.

**Table 3:** Relationship between internet access methods and Web-based health information seeking

Variables		All (N)	Web-based health information seeking, n (%)	*P* value
Apps for smartphones and tablets		1006	891 (88.56)	<.001
Search engines (eg, Google, Bing, and Yahoo)		1091	954 (87.44)	<.001
Social media		901	790 (87.7)	.008
**The most used social media platform**				
	Twitter	358	314 (87.7)	.96
	Instagram	304	266 (87.5)	.96
	Facebook	11	10 (90.9)	.96
	Snapchat	156	134 (85.9)	.96
	Messaging apps	104	93 (89.4)	.96
YouTube		1007	882 (87.58)	.007
Emails to doctors		312	282 (90.4)	.03
Websites		881	785 (89.1)	<.001

Table 4 shows that students mostly use Web-based sources to get health information to read about causes and symptoms of an illness (468/1049, 44.61%), followed by finding information to decide if a visit to the doctor is needed (206/1049, 19.63%). On the contrary, Web-based resources are used least to contact doctors on the Web (49/1049, 4.67%). In addition, most students (684/1049, 65.20%) reported that they actually find health information through Web-based resources, compared with a few who did not (378/1049, 36.03%).

The study revealed that 44.61% (468/1049) of the students used the internet to read about the causes and the symptoms of an illness. Approximately 19.63% (206/1049) of the students seek health information on the Web to decide if they need to consult a doctor, and 18.01% (189/1049) of the students seek Web-based health information before their appointment. The study also showed that 14.58% (153/1049) used the internet to find information after an appointment with a health professional. On the contrary, only 4.67% (49/1049) of the students contact their doctors on the Web. In addition, 65.20% (684/1049) of the students reported finding the desired health information through Web-based resources.

Table 5 describes how students rate the importance of several factors when reading about health information on websites. The study found that the most important factor is the credibility of the information sought (746/1049, 71.11%), followed by the accuracy of the information found (674/1049, 64.25%), and how easy the website is to read (625/1049, 59.58%). On the contrary, the students rated the interactivity of a website as the least important factor (418/1049, 39.84%), followed by the appearance and layout of the website as the second least impactful factor (263/1049, 25.07%) to determine the importance when reading about health information on websites.

The study found that the most important factor for reading health information on the Web chosen by the students was the credibility of the information sought (746/1049, 71.11%). Other important factors include the accuracy of the information found (674/1049, 64.25%) and how easy the website is to read (625/1049, 59.58%). On the contrary, the students rated the interactivity of a website as the least important factor (292/1049 27.83%), followed by the appearance and layout of the website as the second less important factor (377/1049, 35.93%).

Table 6 shows the students’ feelings after obtaining health information on the Web. The study found that the most felt emotion to the least was relief (357/1049, 34.03%), confusion (311/1049, 29.64%), stress and anxiety (244/1049, 23.26%), and curiosity (141/1049, 13.44%).

**Table 4:** The frequency of internet use to get health information on the Web (N=1049).

Variables	Values, n (%)			
	Always	Often	Occasionally	Never
Find information to decide if you need to consult a doctor	206 (19.63)	303 (28.88)	436 (41.56)	117 (11.15)
Find health information before a medical appointment	189 (18.01)	284 (27.07)	383 (36.51)	207 (19.73)
Find information after an appointment with a health professional	153 (14.58)	271 (25.83)	357 (34.03)	282 (26.88)
Contact your doctor on the Web	49 (4.67)	111 (10.58)	226 (21.54)	678 (64.63)
Read about causes and symptoms of an illness	468 (44.61)	342 (32.60)	209 (19.92)	41 (3.90)
What is the frequency rate of finding the health information through Web-based resources	209 (19.92)	475 (45.28)	361 (34.41)	17 (1.62)

**Table 5:** Rating the importance of several factors when reading about health information on websites.

Factor	Values, n (%)		
	Not important	Fairly important	Very important
Accuracy	78 (7.43)	295 (28.12)	674 (64.25)
Current Information	83 (7.91)	360 (34.31)	602 (57.38)
Credibility	49 (4.67)	248 (23.64)	746 (71.11)
Comprehensiveness	101 (9.62)	396 (37.75)	547 (52.14)
Ease of understanding	80 (7.62)	341 (32.50)	623 (59.38)
Readability	83 (7.91)	331 (31.55)	625 (59.58)
Confidentially	239 (22.78)	316 (30.12)	482 (45.94)
Interactivity	418.0 (39.84)	333 (31.74)	292 (27.83)
Appearances	263 (25.07)	405 (38.60)	377 (35.93)

**Table 6:** Students’ feelings after obtaining health information on the Web.

Variables	Values, n (%)
Relieved	357 (34.03)
Stressed and anxious	244 (23.26)
Confused	311 (29.64)
Curious	141 (13.44)

Most of the students were relieved after obtaining health information on the Web (357/1049, 34.03%). In contrast, 29.64% (311/1049) of the students reported getting confused after searching health information on the Web. About 23.26% (244/1049) of the students reported feeling stressed and anxious, whereas 13.44% (141/1049) reported feeling curious after retrieving health information on the Web.

Table 7 describes whether the Web-based resources obtained increased the awareness of the students about the topic. Most of the students agreed (508/1049, 48.42%) with the fact that their awareness has increased after seeking health information on the Web.

Most of the students at Kuwait University agree (803, 76.54%) that seeking health information on the Web increased their awareness about the topic that they read. However, only 4.19% (44/1049) disagreed to the fact that seeking Web-based health information increases their awareness.

Table 8 describes how often obtaining health information on the Web has improved the students’ health. Most of the students reported that their health improved occasionally (517/1049, 49.28%) after obtaining the health information on the Web.

Around 49.28% (517/1049) of the students reported that their personal health improved occasionally after using Web-based health information. Meanwhile, 11.91% (125/1049) of them reported that using Web-based health information always improved their personal health. Furthermore, the study showed that 8.48% (89/1049) of the students reported that their health never improved after obtaining health information on the Web.

Table 9 illustrates the binary logistic regression of significant sociodemographic factors associated with Web-based health information seeking. Students aged 21 years and above are 2.391 times more likely to seek health information on the Web than students aged 18 to 19 years. Age was significantly associated with Web-based health information seeking (*P*=.001). In addition, female students are 2.781 more likely to seek health information on the Web than male students. There was also a significant correlation between gender and Web-based health information seeking with a *P* value of <.001. Moreover, students who are not experienced with internet use are 0.528 times less likely to seek health information than students who are very experienced with internet use. This relationship between the level of experience with internet use and health information seeking was significant (*P*=.01). The table was adjusted for all significant variables in the chi-square table. Significant associations were identified as *P*<.05.

Students aged 21 and above are 2.391 times more likely to seek health information on the Web than students aged (18-19) In addition, female students are 2.781 more likely to seek health information on the Web than male students. Moreover, students who are not experienced with internet use are 0.528 times less likely to seek health information than students who are very experienced with internet use. The relationship between age (*P*=.001), gender (*P*<.001), and level of experience with internet use (*P*=.01) and health information seeking were significant.

**Table 7:** Increased awareness about the topic from resources obtained on the Web.

Variables	Values, n (%)
Strongly agree	295 (28.12)
Agree	508 (48.42)
Not sure	207 (19.73)
Disagree	34 (3.24)
Strongly disagree	10 (0.95)

**Table 8:** Frequency of improvement of personal health after seeking health information on the Web.

Variables	Values, n (%)
Always	125 (11.91)
Usually	325 (30.98)
Occasionally	517 (49.28)
Never	89 (8.48)

**Table 9:** Binary logistic regression of significant sociodemographic factors associated with Web-based health information seeking (n=1126).

Sociodemographic factors		Adjusted odds ratio (95% CI)	*P* value^a^
**Age (years)**			
	18-19	Reference	.001
	20-21	1.322 (0.884-1.977)	.001
	More than 21	2.391 (1.496-3.821)	.001
**Gender**			
	Male	Reference	<.001
	Female	2.781 (1.912-4.044)	<.001
**Level of experience**			
	Very experienced	Reference	.01
	Fairly experienced	0.600 (0.421-0.855)	.01
	Not experienced	0.528 (0.528-0.160)	.01

^a^Significance: *P*<.05.

An illustration of the percentage of barriers faced while using the Web for health information is presented in Multimedia Appendix 4. Approximately 46.7% of the students had difficulty in understanding medical terms; however, 7.3% of the students reported the absence of facilitators as the problem that they faced when seeking health information on the Web.

The percentage for what makes the Web preferable in seeking health information is shown in Multimedia Appendix 5. It was found that about 83.74% (948/1132) of the students search for health information on the Web out of interest and curiosity. On the contrary, 22.34% (253/1132) of the students use Web-based resources owing to a lack of medical insurance.

The percentage of students’ usage of information found on the Web is illustrated in Multimedia Appendix 6. The percentage of those who used Web-based health information to make lifestyle changes was 68.63% (777/1132). Meanwhile, 22.08% (250/1132) of the students changed their medication without discussing it with their doctor after reading health information on the Web.

## Discussion

This study found an overall high prevalence of Web-based health information seeking, with a variation that was noted according to gender, year of study, and faculty. Other variables were identified that affect the association, and the effect on attitudes was explored.

### Prevalence of Web-Based Health Information Seeking Among Students at Kuwait University

The findings of the study revealed that most of the students 86.21% (976/1132) reported using Web-based resources to obtain health information, whereas 13.86% (157/1132) of the students have never used Web-based resources for health information. The same results were found in several studies conducted in Kuwait, Saudi Arabia, and Qatar that showed the prevalence to be 62.9%, 58.4%, and 71.1%, respectively [10-12]. In the United States, a study reported the high prevalence of using Web-based resources to obtain health information (61%) [9]. In contrast, a study conducted in Europe reported that the prevalence of using the Web to retrieve health information was 41.4%, 38.7%, and 33.5% in Denmark, the Netherlands, and Sweden, respectively. The same study also showed the prevalence to be 11.7%, 13.5%, 14%, and 15.3% in Greece, Spain, Portugal, and France, respectively [13]. The increased prevalence of using the Web to retrieve health information could be attributed to the fact that the Web is becoming more easily accessible and affordable to everyone, in addition to the availability of personal devices and campus Wi-Fi, which is provided to the public with minimal charge.

### Association Between the Sociodemographic Factors of the Students and Their Health Information–Seeking Patterns

This study found that most of the Web-based health seekers were aged older than 21 years, whereas a minority of the seekers were within the younger demographic. A study conducted in Portland also found that young adults aged between 18 to 29 years use Web-based resources to seek health information [14]. In addition, it was found that people aged less than 30 years search health information on the Web in a study conducted in France [13]. With regard to the academic year, this study showed that students in their senior years sought health information more than students in their junior years. An increase in searching the Web for health information was noticed in the older age group (21 years and older) compared with the younger age group (18 to 19 years). These trends could be attributed to the increased independence as the students grow older, because they are more likely to act without consulting any family member about their condition. This necessitates prior knowledge about the health situation, which can be obtained through Web-based health information seeking.

In addition, the results of this study showed that non-Kuwaiti Arab students (n=117) sought health information on the Web more than Kuwaiti students (n=898). This has been contradicted in a study conducted in Kuwait, in which Kuwaiti participants (n=58) were more likely to use Web-based resources to obtain health information than non-Kuwaitis (n=71) [10]. The higher percentage of Web-based health information seeking among non-Kuwaitis could be due to the lack of medical insurance and due to the fact that a recent increase in health service expenses was applied to expatriates.

With regard to gender, this study shows a positive association that was significant. The results of several studies that were conducted in Egypt and France were consistent with this study in that there was a significant association between gender and health information seeking on the Web [7,13]. In addition, this study also showed that female students seek health information more than their male counterparts with an adjusted odds ratio of 2.781. This is supported by a study conducted in France, which showed that the odds of female students seeking health information on the Web are 1.64 times higher than the male students [13]. These results were consistent with a study conducted in Nigeria [15]. In contrast, the study that was conducted in Kuwait contradicted this result, indicating that Web-based health information seeking was higher in males (n=96) than in females (n=33) [10]. The difference in the proportion of health information seeking among females and males could be explained by the fact that women are more curious and concerned about their health, in addition to the increased stigma about female reproductive health in Kuwait.

The findings of this study show that students who majored in faculties of Science were more likely to seek health information than those who majored in faculties of Literature. These results were supported by a study done in Nigeria, which found that students in Science faculties use Web-based resources more than students in non-Science faculties [16]. In this study, we found that students in the Faculty of Pharmacy sought health information on the Web the most and students in the Faculty of Education were the least to seek health information on the Web. Additionally, the study conducted in Nigeria showed that the highest percentage of Web use for health seeking was among Health Sciences students, whereas the lowest proportion of Web use to seek health information was among students of the Faculty of Education [16]. This could be justified by the fact that medical, dentistry, and pharmacy students in particular are more likely to search for health information on the Web as it is related to their majors; therefore, Web-based resources are used to learn about specific health information for academic purposes, rather than being sought to improve health.

With regard to the main device used to obtain health information on the Web, this study found that most students used the smartphone, which can be explained by the unparalleled accessibility that mobile phones offer, which is access to Web-based resources. This is supported by a study conducted in Kuwait in 2017, which showed that 99.5% of university students reported owning or using a mobile phone [17].

In the study, it was found that the most frequently used type of internet on campus was mobile data (n=644) and the least was internet cable (n=3). This result is supported by the study in Ghana, which reported that the main type of internet used among students to seek Web-based health information was mobile data (n=268) and the least was local area network (n=33) [1].

This research also showed that the primary source of internet used by students to obtain health information was personal devices (n=874), compared with the study conducted in Ghana, which showed campus laboratories and Wi-Fi to be the primary source of internet used (n=268) [1]. These findings could be explained by the fact that young adults in Kuwait are up to date with the advancements in technology and so most of them own a mobile phone, which makes it more convenient to use mobile data.

The findings of this study showed that students who are more experienced with Web use (576/650, 88.6%) rely on Web-based resources to seek health information more than those who are fairly experienced (449/537, 83.6%) or inexperienced (15/19, 79%). In a study conducted in the United States in (2011-2014), it was found that throughout the 4 years of this study, participants who had a high level of Web experience were more likely to seek health information than those who had no level of Web experience [18]. This result was consistent with another study conducted on adolescents from the United Kingdom and the United States, which found that a significant number of participants who had previous Web experience were actively involved in Web-based health information retrieval [19]. This difference in proportion may be explained by the difficulties faced by inexperienced individuals when seeking health information on the Web, compared with those with prior experience with Web use.

### Influence of the Information Found on the Web on the Attitudes of Students at Kuwait University

In this study, it was found that most of the students use the health information that they sought on the Web to make lifestyle changes and discuss this information with their doctors, and only a few of them used the health information to change their medication without discussing it with their doctors. Similar results were found in a study conducted in Ghana; it was found that most of the students used the health information to make lifestyle changes and a few of them used it to make, cancel, or change their appointments with their doctors [19]. In addition, a study that was conducted in India found that most of their respondents shared the health information with friends and family and that the major factors that affect Web-based health information seeking were behavioral and habitual changes [20].

The findings of this study also revealed that most of the students felt relieved after reading about health information on the Web (357/1049, 34.03%).

Moreover, most of the students agreed (508/1049, 48.42%) with the fact that their awareness was increased after seeking health information on the Web. In addition to that, it was found that most of the students reported that their health improved after reading health information on the Web (517/1049, 49.28%). This result was supported by the findings in a Ghana study that revealed that the students’ health conditions improved after seeking health information on the Web [1]. This can be attributed to the fact that finding health information on the Web increases the individual’s awareness about their health, which can lead to making better informed health decisions.

With regard to the barriers that the students face while searching for health information on the Web, the results showed that the dominant barriers were understanding medical terms and a lack of Web searching skills. This dominance of the difficulty in understanding medical terminology could be explained by the fact that most of the students have no knowledge of medical terminology and most of the websites used for health information seeking use medical terms. On the contrary, the lack of skill needed could be attributed to a lack of proper training in effective Web use for health-seeking purposes.

Finally, this study focused on the factors that made the Web preferable for seeking health information. Interest and curiosity were the dominant factors 83.74% (948/1132) that affected the students to consider Web-based health information preferable. This can be explained by the innumerable amount of health information accessible to students through these recourses. The least reported factor to make the Web a preferable source of health information is lack of medical insurance. This can be due to the fact that health care is provided to Kuwaiti people free of charge, and the sample mostly consists of Kuwaiti students.

### Conclusions

The study concluded that there is a large prevalence of Web-based health information seeking among students at Kuwait University. In addition, sociodemographic factors, for example, age, gender, faculty, year of study, primary source of internet, and level of experience with the Web, were significant correlates to Web-based health information seeking among the students. The study also found that more than half of the students who seek health information on the Web made lifestyle changes or discussed the information found with their doctor, although this finding was not statistically significant, possibly because of chance. There is an increasing trend in Web-based health information seeking among college students worldwide. Therefore, people should become more aware about the quality of the information sought, and doctors should educate the people to increase that awareness.

### Strengths

Few studies have been conducted in Kuwait to study the association between seeking health information on the Web and its influence on the population. Another strength is the appropriate sample size, covering 8 faculties from both Science and Literature. Moreover, the 8 faculties were randomly selected from the available faculties of Kuwait University. Nevertheless, some limitations were identified.

### Limitations

The limitations of the study are as follows: (1) the fact that university students may not be totally representative of all health information seekers as it is a young population and not all age groups are included in the study, and therefore, the study does not include people from different educational backgrounds and age groups; (2) in addition, the study was conducted in Kuwait University, in which most of the students are Kuwaiti, unlike the actual population in Kuwait, which consists of a majority of non-Kuwaiti individuals; and (3) most of the study population consisted of females, which could have affected the results with regard to gender.

### Recommendations

Future studies should be conducted to (1) assess the level of awareness of Web users when it comes to the reliability and integrity of websites that are used to seek health information, (2) increase the awareness of the users with regard to trusted websites, and (3) include cultural factors and their effects on Web-based health information–seeking behaviors of users, including students, and factors that affect Web-based health information seeking, such as having a chronic illness or having a family member affected by an illness.

## Appendix

Multimedia Appendix 1 The rejection rate.

Multimedia Appendix 2 Informed consent form, human subjects form, and ethical portfolio.

Multimedia Appendix 3 Data collection instruments (questionnaires).

Multimedia Appendix 4 Percentages of barriers faced while using the internet for health information.

Multimedia Appendix 5 Percentage of what makes the internet preferable for seeking health information.

Multimedia Appendix 6 Percentage of students’ usage of information found on the Web.
