# Supplementary figures and images for: Web-Based Health Information Seeking Among Students at Kuwait University: Cross-Sectional Survey Study
Source: JMIR Form Res. 2019 Oct 31;3(4):e14327. doi: 10.2196/14327 (PMC6914278; doi:10.2196/14327)

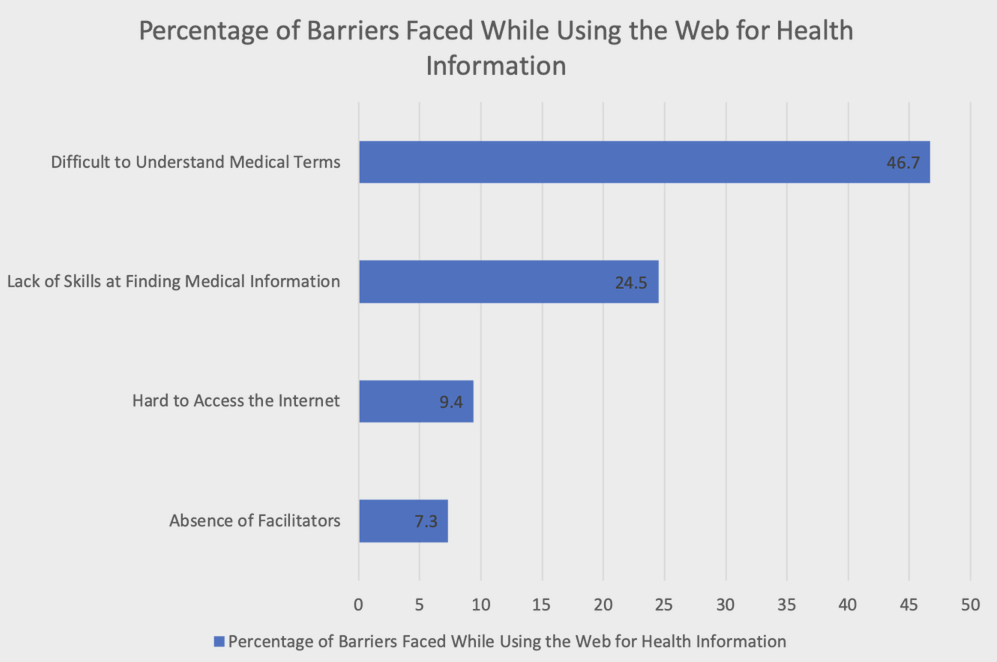

Supplement: Multimedia Appendix 1 [file formative_v3i4e14327_app.png]
