# Supplementary material for: Web-Based Health Information Seeking Among Students at Kuwait University: Cross-Sectional Survey Study
Source: JMIR Form Res. 2019 Oct 31;3(4):e14327. doi: 10.2196/14327 (PMC6914278; doi:10.2196/14327)
Supplement: Multimedia Appendix 2 [file formative_v3i4e14327_app.pdf]

Appendix A: Informed Consent Form, Human Subjects Form, and Ethical Portfolio

جامعة الكويت
كلية الطب
قسم طب المجتمع

إقرار مستنير للطلاب المشاركين بالدراسة

عنوان الدراسة: البحث عن المعلومات الطبية عبر المصادر الالكترونية: دراسة على طلبة جامعة الكويت

الهدف من هذه الدراسة هو: معرفة وجهات نظر طلبة جامعة الكويت باستخدام المصادر الالكترونية للبحث عن المعلومات الطبية. لذلك، ينبغي عليك اختيار الإجابة الأنسب للاسئلة، علما بأن إجابتك سوف تكون سرية وسوف تستخدم لأغراض البحث فقط.

الإجراءات المتبعة في هذه الدراسة تتضمن التالي:
- **الإجابة على ٣١ سؤال من خلال هذا الاستبيان والذي يستغرق حوالي ٥-١٠دقيقة تقريبا لإكماله.**

الفئة العمرية المطلوبة للمشاركين في الدراسة هي من 18 سنة إلى 25 سنة.
لن تتعرض للمخاطر إذا شاركت في هذه الدراسة. مشاركتك سوف تزيد من معرفتنا بهذه القضية الهامة. جميع المعلومات التي سيتم جمعها ستظل سرية واسمك سوف يحفظ في ملف منفصل عن البيانات الأخرى ولن يستخدم في الدراسة. المشاركة في هذه الدراسة اختيارية وليست اجبارية، ولك كامل الحرية في الموافقة او عدم الموافقة في المشاركة علما بأن عدم الموافقة لن تؤثر على وضعك/ تحصيلك الأكاديمي. لك كامل الحرية بالتراجع/ الانسحاب عن المشاركة في هذه الدراسة في أي وقت.

<u>يرجى وضع علامة (✓) أدناه إذا كنت ترغب في المشاركة أو الامتناع عن القيام بذلك:</u>

أرغب بالمشاركة **اسم المشارك في الدراسة:** ____________________

لا أرغب بالمشاركة **توقيع المشارك في الدراسة:** ____________________

نشكر لكم حسن تعاونكم.

اسم الطالب: رباب أسيري، نور العلي، شهد مبارك، حوراء ملاح، فجر بوكبر، حسن أشكناني
اسم المشرف على الدراسة: د.علي بوعباس، أ. عبدالله المجرن **التاريخ: ١٨-٣-٢٠١٨**

Kuwait University
Faculty of Medicine
Department of Community Medicine

Informed Consent Form for Students

Title of the Project: Online Health Information Seeking Among Kuwait University Students

The aim of this study is to explore the students' opinions regarding the use of online resources for health purposes at Kuwait University. You are therefore required to answer the questions below to the best of your abilities. Your responses will be anonymous and will be only used for research purposes. Thank you!

The procedures involved in this study include:

- **Answering 31 questions on this questionnaire, which should only take you about 5-10 minutes to be completed.**

The desired age group of the participants is from 18 years to 25 years.
There are no risks to you if you participate in this. Your participation will increase knowledge about this important issue. All information collected will remain confidential. Your name will be kept in a file separate from the other data and will not be used in the study. There is no obligation or compulsion for you to participate, and you have the freedom to agree or not agree to participate. This will not have any effect on your academic standing or your grades. You may withdraw from the research at any time. This research does not include any medical experiments, taking biological samples.

Please indicate (✓) below if you wish to participate or decline to do so:

☐ **I wish to participate.** **Name of participant: ____________**

☐ **I do not wish to participate.** **Signature of participant: ____________**

Thank You for Your Cooperation . . .

Student names: Rabab Asery, Noor Al-Ali, Shahad Mubarak, Hawraa Mallah, Fajer Bokubar, Hasan Ashkanani

Supervised by: Dr. Ali Buabbas, Mr. Abdullah Al-Majran **Date: 18/3/2018**

Faculty of Medicine

Department of Community Medicine

Ethics Portfolio

Online Health Information Seeking Among Kuwait University Students

18/3/2018

Rabab Asery, Noor Al-Ali, Shahad Mubarak,
Hawraa Mallah, Fajer Bokubar, Hasan Ashkanani

Supervising Faculty Member: Dr. Ali Buabbas

Project Assistant: Mr. Abdullah Al-Majran

Project required for course: Community Medicine rotation

Ministry of Health
The Standing Committee for
Coordination of Health and
Medical Research

وزارةالصحة

اللجنة الدائمة لتنسيق
البحوث الطبية والصحية

البيانات الرئيسية لمشروع بحث للعرض على اللجنة

تاريخ تقديم الطلب: [18 / 3 / 2018] رقم البحث : السنة:2018

الباحث الرئيسي: د. علي بوعباس Faculty Supervisor:<br>Dr. Ali Buabbas<br>هاتف نقال:99809570 فاكس: 25338948	باحثون مشاركون: رباب أسيري، نور العلي، شهد مبارك، حوراء ملاح، فجر بوكبر، حسن أشكناني<br>Students: Rabab Asery, Noor Al-Ali, Shahad Mubarak, Hawraa Mallah, Fajer Bokubar, Hasan Ashkanani
المنطقة/ المستشفى /القسم / الادارة<br>انظر الى المرفق "ملخص خطة البحث"	مدة مشروع البحث : مدة جمع البيانات أسبوعين ابتداء من [2018/3/29 الى 2018/4/14]

عنوان البحث المقترح / Research Title : البحث عن المعلومات الطبية عبر المصادر الالكترونية: دراسة على طلبة جامعة الكويت
Online Health Information Seeking Among Kuwait University Students

أهداف البحث / Objectives :
See "Study Objectives" in the attached Ethics Portfolio. انظر الى المرفق "ملف الأخلاقيات" تحت عنوان "أهداف البحث"

ملخص خطوات البحث / Research Plan / Protocol (1):
See the attached "Summary of Research Plan" انظر الى المرفق "ملخص خطة البحث"

النتائج المتوقعة / Expected Outcome :
See "Expected Outcomes" in the attached Ethics Portfolio. انظر الى المرفق "ملف الأخلاقيات" تحت عنوان "الفوائد والنتائج المتوقعة"

إقرار الموافقة علي المشاركة بالبحث (يرجي إرفاق نسخة من الإقرار باللغة العربية والإنجليزية) Informed Consent (2):
See "Informed Consent Forms" in the attached Ethics Portfolio. انظر الى المرفق "اقرار الموافقة "

إجراءات حقوق المرضي لضمان سرية المعلومات والخصوصية:<br><br>See “Confidentiality” in the attached Ethics Portfolio. انظر الى المرفق "ملف الأخلاقيات" تحت عنوان "الخصوصية"

الميزانية التقديرية :	جهة التمويل الخارجية :

الموافقة علي تقديم البحث		
الباحث الرئيسي:	رئيس القسم :	مدير المستشفى / الإدارة :

ملاحظات:

يتم استلام هذا النموذج من قسم البحوث بإدارة التخطيط والمتابعة، ويقوم الباحث المتقدم بعد استيفاء البيانات بالنموذج وإرفاق الوثائق المذكورة أعاله بأرقام(1) ، (2) بتسليمهم للسيدة / رئيس قسم البحوث بإدارة التخطيط والمتابعة حيث سيرسل للجنة الدائمة والتي بواسطتها سيبلغ المتقدم برأي اللجنة لاحقا.
هواتف قسم البحوث : مباشر 42836842 بدالة : 42888244 / 42888838 داخلي: 4828 / 4634
فاكس: 42836382 يكتب علي الفاكس عبارة ((لعناية السيدة / رئيس قسم البحوث خاص بالبحث رقم ....))

This form is to be submitted to the HSC Ethics Committee for Student Research

Summary of Research Plan

Date: 18/3/2018

Faculty and Department:
Faculty of Medicine- Department of Community Medicine

Investigator's names and designation:
Faculty supervisor: Dr. Ali Buabbas

Kuwait University Faculty of Medicine Students:

Rabab Asery

Noor Al-Ali

Shahad Mubarak

Hawraa Mallah

Fajer Bokubar

Hasan Ashkanani

Project Title: Online Health Information Seeking Among Kuwait University Students

Summary of the Study:
With the recent development in technology, resources are more accessible than ever. With this accessibility, comes the ease of finding health information using online resources. This study aims to assess the use of these online resources to seek health information among Kuwait university students, and how that affect seeking healthcare and the attitude of students towards the information found online. This study will be done in a cross-sectional manner, in which a sample of 800 students aged 18 years and above from all nationalities and from both genders will be taken, aiming to start on march 29th and spanning for two weeks. It will include handing out questionnaires and then analyzing the data obtained from the students.

Detailed Research plan:
Please see the attached Ethics Portfolio for details.

عنوان البحث المقترح: البحث عن المعلومات الطبية عبر المصادر الالكترونية: دراسة على طلبة جامعة الكويت

أسماء الأماكن المحددة التي سيتم جمع البيانات فيها:

كلية الطب
كلية الصيدلة
كلية طب الأسنان
كلية العلوم
كلية التربية
كلية العلوم الإدارية
كلية الشريعة
كلية الحقوق

Project Title: Online Health Information Seeking Among Kuwait University Students

Names of specific sites to be accessed for data collection:

- **Faculty of Medicine**
- **Faculty of Pharmacy**
- **Faculty of Dentistry**
- **Faculty of Science**
- **Faculty of Education**
- **Faculty of Business Administration**
- **Faculty of Sharia and Islamic Studies**
- **Faculty of Law**

Human Subjects Form

Title: Online Health Infromation Seeking Among Kuwait
Study Objectives:

-To determine the prevalnce of Kuwait University students using online retrieval of health information.
-To demonestrate how Kuwait University students are influenced by the health information found online.
-To assess the use of the internet for health purposes among Kuwait University Students
-To identify any association between sociodemographic of students (age, information-background, gender) and their health information seeking pattern.
-To explore how the attitudes of Kuwait University students are affected by seeking health information online.

Human Subjects

Yes	No	
√		**Are human subjects to be enrolled?**

If yes, from which population will they be identified?

- [ ] **General population, adults (specify: ________________________ ____________)**
- [ ] **General population, minors (specify: ______________________ ____________)**
- [ ] **Patients, outpatient setting (specify: ________________________ ____________)**
- [ ] **Patients, inpatient setting (specify: _________________________ ______)**
- [ ] **Other (specify: Students aged 18 years and above)**

Estimated Sample size: 800

Methods

The methods of the study include the following:

- [√] **Questionnaire**
- [ ] **Screening/diagnostic procedures/tests (specify: ______________ ______)**

☐ **Physical examination (specify: ________________________________ ____________)**

☐ **Other (specify: ____ ____________)**

Risks

Does the research pose any risk to participants in the following categories?

Yes	No	
☐	√	**Risks to social or psychological well-being? (specify: ____________________)**
☐	√	**Risks to physical well-being? (specify: ____________________)**
☐	√	**Conflict with local religious or cultural beliefs? (specify: ____________________)**
☐	√	**Risks over and above routine clinical care? (specify: ____________________)**
☐	√	**Risks from invasive procedures? (specify: ____________________)**
☐	√	**Other risks? (specify: ____________________)**

Benefits

List the benefits the participant and/or medical science will receive from this study -Knowing the effects of online health information seeking on healthcare, the attitude of patients towards healthcare providers, and effect of information found online on the attitudes of the patients.

Yes	No	
√	☐	**In your opinion, do the benefits outweigh the risk?**

Confidentiality

Yes	No	
√	☐	**Is confidentiality assured in this study?**

If so, how?

√ **Anonymous data collection (name or identifying information not**

☐ **obtained)**

☐ **Name taken, but stored in a separate database from other study databases, which**
identify participants using ID numbers only.

☐ **Study forms with identifiers are accessible to study personnel only, and are stored**
in locked cabinets.

☐ **Other precautions (specify: ______________________________________)**

Informed Consent

Yes	No	
√	☐	**The Informed Consent form is prepared in English and Arabic**
√	☐	**Is it clearly stated that participation is voluntary, that no negative consequences will result if the prospective participant does not choose to participate, and that the participant may withdraw from the study at any time without any negative consequences?**

Students' Statement

Project Title: Online Health Information Seeking Among Kuwait University Students.
We affirm that we will respect the following guidelines:

1) **The students and the tutor alike should not submit any part of the research for publication or presentation at conferences without the knowledge and consent of all the students and of the project tutor.**
2) **If the students would like to publish their project, they should notify their tutor who has the following options:**
   a) **The right to further guide the students until the project is published. In this case, it is expected that the tutor designates himself/herself as a senior author (the last and the corresponding author). The tutor should not designate himself/herself as the first author.**
   b) **The right to choose not to participate in publishing the project, with a specification that he/she will be acknowledged in any publications. In this case, tutor should give the students a written, signed statement showing his/her unwillingness to participate, but with the specification that he/she should be**

acknowledged in any publications.

c) **The right to choose not to participate in publishing the project and to remove his/her name from any publications either as a co-author or in the acknowledgment. In this case, tutor should give the students a written, signed statement showing his/her unwillingness to participate.**

3) **If the tutor would like to publish the project and only some of the students are interested in publishing, the tutor should ask the students who want to be exempted from the publication for a written statement that shows their unwillingness to participate in publishing the project, or of being a co-author on the publication. If some students are not responding to the request of the tutor, the tutor should ask the Department Chairman, who will also communicate with the students to request the statement of non-participation. If the students do not respond to the communication from the Department Chairman, their names should not be included in the paper. The authorship order in this case should follow the guidelines in Item No. 2(a) above.**

4) **To avoid conflicts regarding order of authorship between the students and tutor or between the students themselves, the tutor should obtain from the students an agreement on the title and order of authorship before the preparation of the manuscript. The agreement should be signed by all students and the tutor, and kept with the tutor. This should be considered only if the tutor is willing to participate in the publication of the project according to the authorship guidelines in items No. 2(a) above. In case the tutor chooses not to participate, then it is up to the students to make such arrangements.**

5) **The above policy should also be followed in presenting data in conferences. In the exceptional circumstances when the tutor is the only member of the research team attending the conference and the conference organizers request the presenting author to be the first author, it is possible for the tutor to designate himself/herself as the first author. In such case, he/she should inform the students about the request of the conference organizers in advance.**

Rabab Asery ______________________________

Noor Al-Ali ______________________________

Shahad Mubarak ______________________________

Hawraa Mallah ______________________________

Fajer Bokubar ______________________________

Hasan Ashkanani ______________________________

Date: 18/03/2018
